# Effect of A/T/N imaging biomarkers on impaired odor identification in Alzheimer's disease

**DOI:** 10.1038/s41598-020-68504-2

**Published:** 2020-07-14

**Authors:** Min Seok Baek, Hanna Cho, Hye Sun Lee, Jae Hoon Lee, Young Hoon Ryu, Chul Hyoung Lyoo

**Affiliations:** 1 Department of Neurology, Gangnam Severance Hospital, Yonsei University College of Medicine, Seoul, South Korea; 2 Biostatistics Collaboration Unit, Yonsei University College of Medicine, Seoul, South Korea; 3 Department of Nuclear Medicine, Gangnam Severance Hospital, Yonsei University College of Medicine, Seoul, South Korea

**Keywords:** Neuroscience, Neurology

## Abstract

Odor identification ability may serve as an important diagnostic biomarker in Alzheimer’s disease (AD). The aim of the study is to investigate the contribution of A/T/N neuroimaging biomarkers to impaired odor identification ability in the Alzheimer’s disease spectrum. In 127 participants, we compared A/T/N neuroimaging biomarkers between normosmia and hyposmia groups, and performed correlation analysis between the biomarkers and Cross-Cultural Smell Identification Test (CCSIT) scores. Additionally, path analysis for odor identification ability was performed using cognitive function as a mediator. In between-group comparison, individuals with hyposmia showed higher frequency of amyloid-β (Aβ) positivity, and lower neuropsychological test performance than those with normosmia. After correction for covariates including total cognition scores, there was no difference in the Aβ or tau burden between the normosmia and hyposmia groups, and no correlation between CCSIT scores and Aβ or tau burden. Meanwhile, cortical volumes in the lateral and medial temporal cortices were smaller in the hyposmia group and decreased with the worsening of CCSIT scores. Path analysis showed that only neurodegeneration had a direct effect on odor identification, while Aβ and tau burden contributed to odor identification with the mediation of cognition. In the Alzheimer’s disease spectrum, impaired odor identification ability may be attributable to neurodegeneration rather than the direct effect of Aβ or tau burden.

## Introduction

Odor identification ability is impaired in patients with Alzheimer’s disease (AD)^1^. A step-wise deterioration of odor identification ability with the advancement of cognitive status enabled discrimination of cognitively unimpaired individuals, mild cognitive impairment (MCI), and AD patients^2,3^. Longitudinal studies on MCI patients also showed that poor odor identification ability was associated with cognitive decline^4,5^, and thereby predicted conversion from MCI to AD^4,6^. In postmortem studies, impaired antemortem odor identification ability was associated with the amyloid-β (Aβ) burden in the global cortex^7^, and tau in the entorhinal cortex and hippocampus^7,8^, and was therefore helpful for predicting Aβ-positivity by in vivo positron emission tomography (PET)^9,10^. Additionally, impaired odor identification has been associated with the CSF biomarker for neurodegeneration^11^ and volume atrophy in the medial temporal cortex^12,13^ where tau pathology appears early in AD^14^. However, in these A/T/N biomarkers, there is no information regarding which biomarker has the greatest effect on odor identification.

Olfactory function not only requires the physical activation of diverse olfactory receptor cells by chemical substances, but also includes adaptation process to provide sensitivity and amplification and inhibition to enhance the odor detection. Subsequently, olfactory information is transferred to the cerebral cortex to interact with emotional response and higher olfactory function such as odor identification^15,16^. This process requires memory and naming functions, suggesting that cognitive impairment may directly affect the odor identification ability^17,18^. Moreover, the brain regions related to the central olfactory pathway, such as the entorhinal cortex, amygdala, and hippocampus overlap with the regions most vulnerable to pathological changes in AD^19–21^. Therefore, it remains unclear whether AD pathology directly effects odor identification ability, or if it is mediated by cognitive dysfunction.

In this study, we sought to investigate the relationship between odor identification ability and each A/T/N imaging biomarker. We additionally investigated whether cognitive dysfunction mediates the association between the biomarkers and impaired odor identification.

## Results

### Demographic characteristics

Detailed demographic characteristics of the study participants are summarized in Table 1. Out of 127 participants, 72 participants showed normosmia and 55 showed hyposmia. The ApoE ε4 allele was more frequently found in the hyposmia group than the normosmia group. The hyposmia group was older and more likely to be Aβ-positive and dementia status than the normosmia group. Likewise, the hyposmia group showed worse global cognition as measured by MMSE score, CDR-SB, and total cognition score and worse cognitive domain functions including memory, language and related, visuospatial, and frontal/executive functions.

**Table 1:** Baseline demographic characteristics cognitive function between normosmia and hyposmia groups.

	Aβ ±			Aβ +		
	Normosmia	Hyposmia	*P *value	Normosmia	Hyposmia	*P *value
N	72	55		25	34	
Sex (M:F)	21:51	22:33	0.159	8:17	12:22	0.792
Age (years)	67.2 ± 9.0	75.4 ± 8.7	**<** **0.001**	70.7 ± 9.3	76.9 ± 8.0	**0.008**
Education (years)	11.0 ± 4.3	11.2 ± 5.2	0.797	10.8 ± 3.7	11.1 ± 5.4	0.840
CCSIT	9.6 ± 1.2	4.9 ± 2.1	**<** **0.001**	9.3 ± 1.1	4.3 ± 2.2	**<** **0.001**
ApoE ε4+ (%)	13 (18%)	20 (36%)	**0.020**	16 (64%)	18 (53%)	0.396
CU/MCI/DEM (%)	42/44/14%	18/35/47%	**<** **0.001**	12/56/32%	9/29/62%	0.073
Amyloid positivity	25 (35%)	34 (62%)	**0.002**	25 (100%)	34 (100%)	n.a
Neuropsychological tests						
MMSE	26.8 ± 2.9	22.7 ± 5.1	**<** **0.001**	25.7 ± 3.8	20.9 ± 5.1	**<** **0.001**
CDR-SB	1.1 ± 1.4	3.0 ± 2.1	**<** **0.001**	2.0 ± 1.7	3.7 ± 2.0	**0.005**
Total cognition score	153.7 ± 38.3	116.7 ± 47.6	**0.045**	128.6 ± 42.1	104.3 ± 39.9	**0.033**
Memory	55.9 ± 22.1	36.3 ± 23.6	**0.010**	41.7 ± 21.7	29.9 ± 18.5	0.108
Language and related	22.1 ± 3.8	19.0 ± 5.4	**0.003**	20.7 ± 4.7	17.7 ± 5.3	**0.033**
Visuospatial	30.5 ± 6.6	26.4 ± 9.5	**0.040**	27.0 ± 9.5	25.4 ± 9.7	0.298
Frontal/executive	35.4 ± 11.3	25.9 ± 13.1	**0.005**	29.5 ± 11.2	22.2 ± 11.0	**0.028**
Attention	9.9 ± 2.1	9.2 ± 2.6	0.270	9.6 ± 2.3	9.0 ± 2.1	0.187

Data are presented as mean ± SD.

Bold values represent statistically significant *P* value of < 0.05.

*CCSIT* Cross-Cultural Smell Identification Test, *CU *cognitively unimpaired, *MCI* mild cognitive impairment, *DEM* dementia, *ApoE* apolipoprotein E, *Aβ+/−* Aβ-positivity, *MMSE* Mini-Mental State Examination, *CDR-SB* Clinical Dementia Rating sum-of-boxes, *n.a* not applicable.

**Figure 1:**
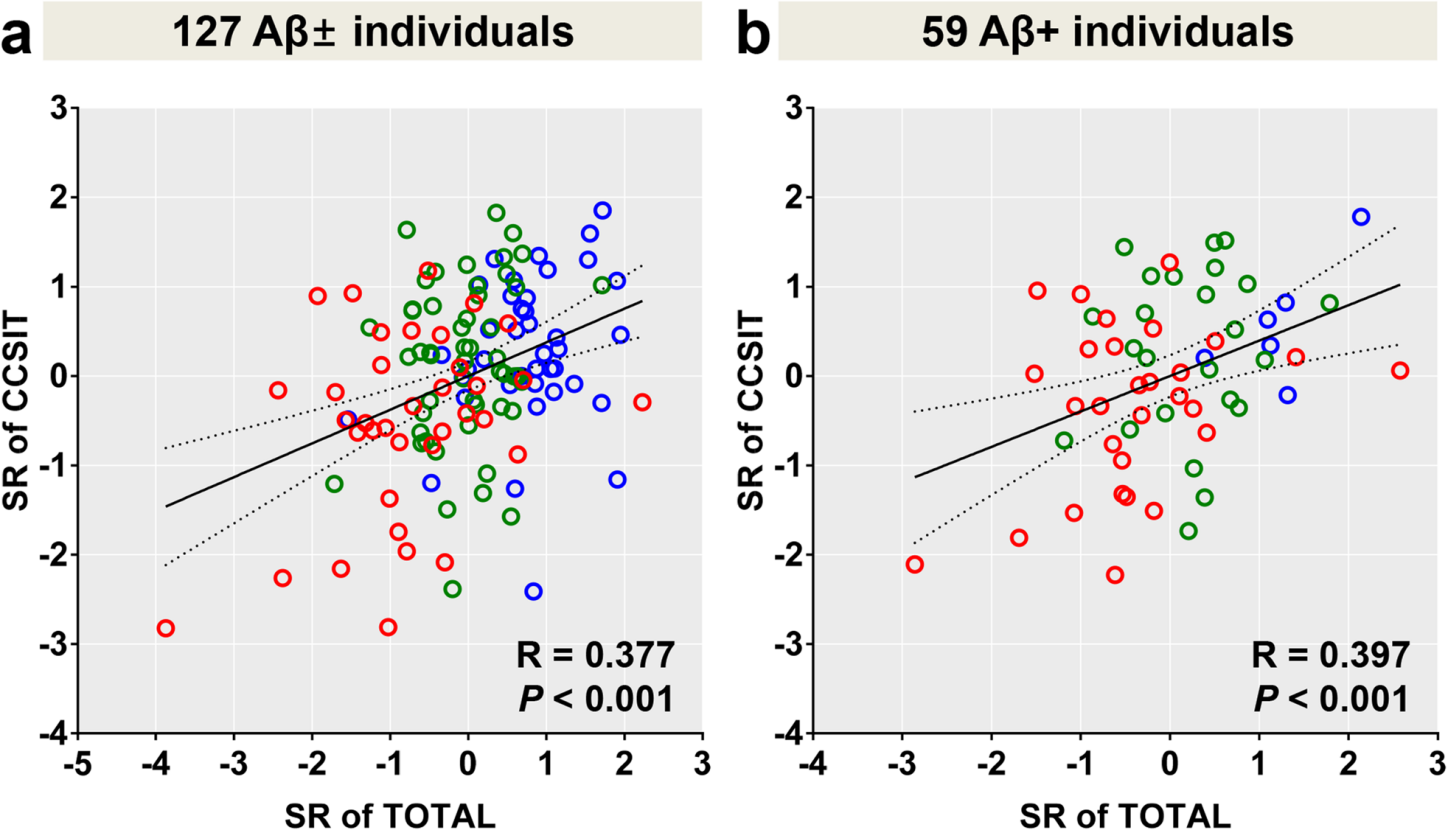
Correlation between the total cognition scores and CCSIT scores. Correlation analysis was performed after adjusting for age, sex, presence of ApoE ε4 and years of education in all 127 participants (**a**), and 59 amyloid-β positive participants (**b**). Blue dots represent CU, green dots MCI patients, and red dots DEM. Abbreviations: Aβ+ /− = Aβ-positivity, SUVR = standardized uptake value ratio, CU = cognitively unimpaired; MCI = mild cognitive impairment; DEM = dementia, ApoE = apolipoprotein E, SR = Standardized residual, CCSIT = Cross-Cultural Smell Identification Test.

In 59 Aβ-positive individuals, the hyposmia group was still older than the normosmia group, and showed worse global cognition and worse cognitive domain functions including language and related and frontal/executive functions. In addition, Cross-Cultural Smell Identification Test (CCSIT) scores correlated with total cognition scores (Fig. 1).

### Comparison of A/T/N biomarkers between the normosmia and hyposmia groups

**Figure 2:**
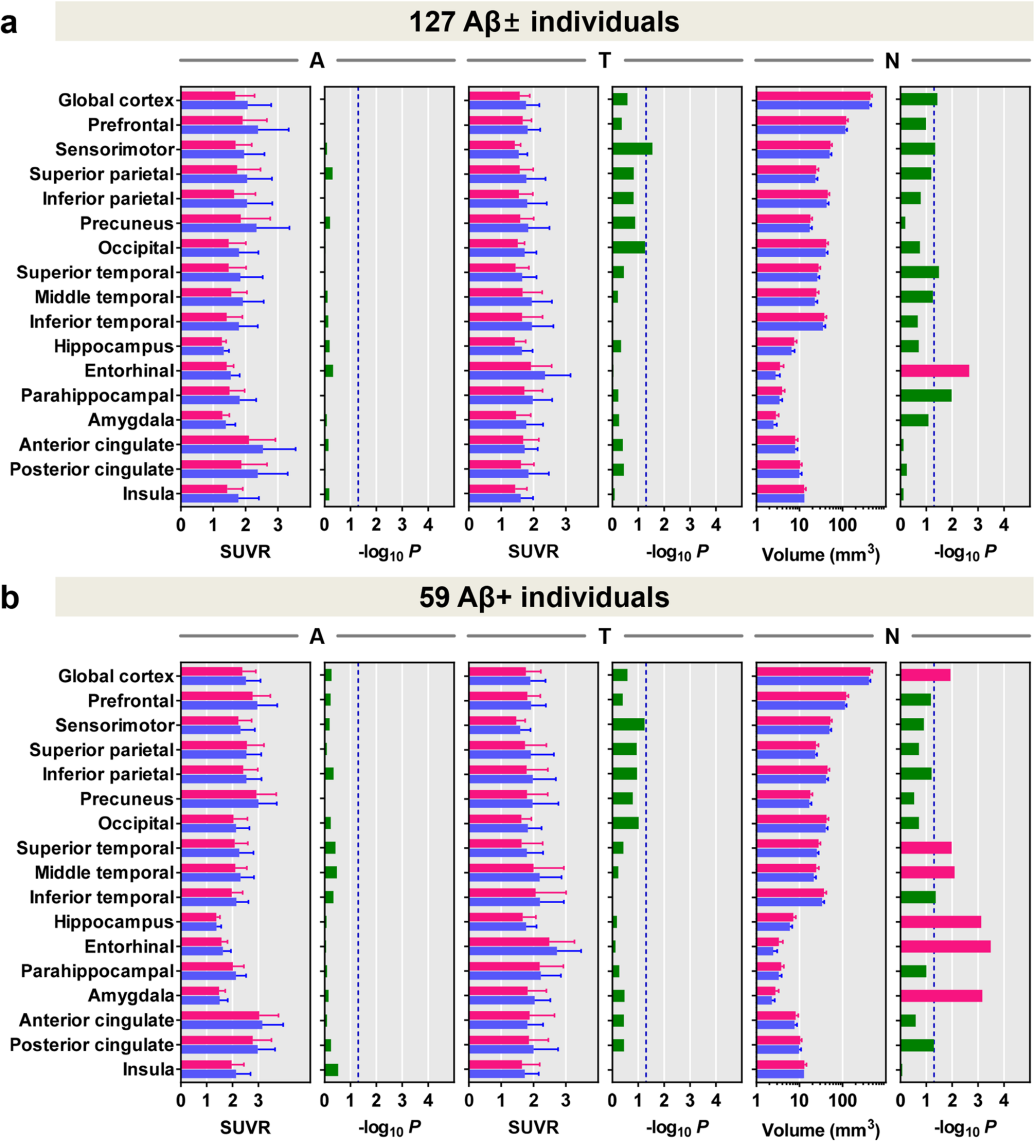
Comparison of ^18^F-florbetaben, ^18^F-flortaucipir SUVR and cortical volume between normosmia and hyposmia groups in all participants (**a**), and amyloid-β positive participants (**b**). Regional SUVR values were compared between the two groups after adjusting for age, sex, years of education, presence of ApoE ε4 and total cognition scores. For the comparison of regional cortical volumes between the two groups, total intracranial volume was added to the list of covariates. Data are presented as means (horizontal bars) and standard deviations (error bars) of normosmia (red) and hyposmia (blue) groups. *P*-values for the difference between two groups are expressed as − log_10_ *P*. *P*-values presented with red bars indicate the regions that survived correcting for region-wise multiple comparisons (false discovery rate-corrected *P* < 0.05), and blue dotted lines represent uncorrected *P* < 0.05. Abbreviations: Aβ+ /− = Aβ-positivity, SUVR = standardized uptake value ratio, A = ^18^F-florbetaben SUVR, T = ^18^F-flortaucipir SUVR, N = cortical volume.

After correction for age, sex, years of education, presence of ApoE ε4, and total cognition score in all participants, the normosmia and hyposmia group did not show difference in the regional Aβ and tau burden. Meanwhile, the hyposmia group exhibited smaller volumes in the entorhinal cortex (Fig. 2a). In Aβ-positive individuals, the hyposmia group showed smaller volumes in the global cortex, superior and middle temporal, and entorhinal cortices, amygdala, and hippocampus (Fig. 2b).

### Association between odor identification ability and A/T/N biomarkers

**Figure 3:**
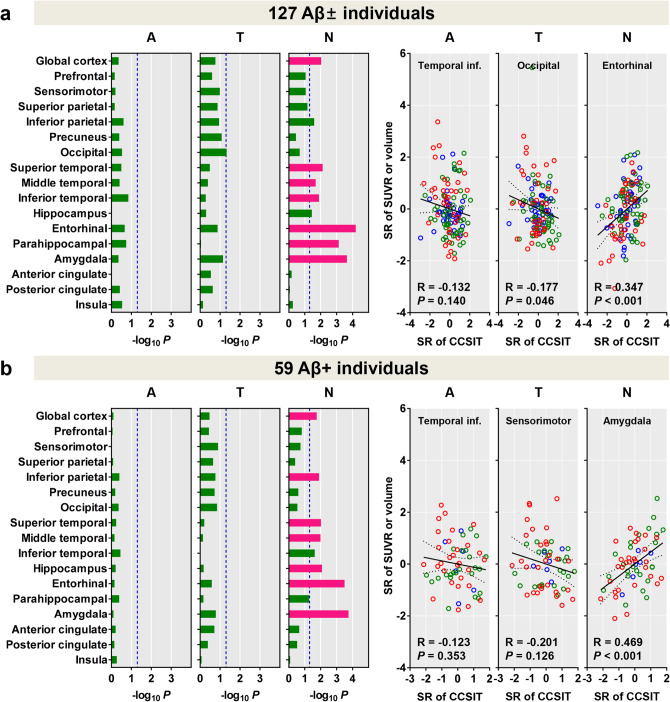
Correlation of CCSIT score with ^18^F-florbetaben, ^18^F-flortaucipir SUVR and cortical volume in all participants (**a**), and amyloid-β positive participants (**b**). We used Pearson’s correlation between the standardized residuals of CCSIT scores and regional SUVR values obtained with multiple linear regression model after adjusting for age, sex, years of education, presence of ApoE ε4 and total cognition scores. A covariate of intracranial volume was additionally adjusted for cortical volume. Horizontal bars represent *P*-values as − log_10_ *P.* Red bars represent the regions that survived correction for region-wise multiple comparisons (false discovery rate-corrected *P* < 0.05), and blue dotted lines represent uncorrected *P* < 0.05. In the right side panel, brain regions showing highest significance in correlation with ^18^F-florbetaben, ^18^F-flortaucipir, and cortical volume are presented respectively. Blue dots represent CU participants, green dots represent MCI patients, and red dots represent DEM. Abbreviations: Aβ+ /− = Aβ-positivity, SUVR = standardized uptake value ratio, A = ^18^F-florbetaben SUVR, T = ^18^F-flortaucipir SUVR, N = cortical volume, SR = Standardized residual, CCSIT = Cross-Cultural Smell Identification Test, CU = cognitively unimpaired; MCI = mild cognitive impairment; DEM = dementia, ApoE = apolipoprotein E.

In contrast to Aβ and tau burden which did not correlate with CCSIT score, low CCSIT score was associated with the atrophy in the global cortex, lateral temporal, entorhinal, and parahippocampal cortices, and amygdala (Fig. 3a). In Aβ-positive individuals, low CCSIT score was related with the atrophy in the global cortex, inferior parietal, superior and middle temporal, and entorhinal cortices, amygdala, and hippocampus (Fig. 3b).

Because there was a correlation between the CCSIT and total cognition scores, we primarily included total cognition score as a covariate to investigate the pure effect of tau and Aβ burden on deterioration of odor identification function independent of worsening global cognition. However, because the tau and Aβ burden also increase with the worsening of cognition, there remains a concern that the correlation between the CCSIT scores and tau and Aβ burden might not be detected. Therefore, we repeated the analysis without controlling for total cognition score and found that CCSIT score correlated with Aβ and tau burden in the widespread cortical regions in all participants (see Supplementary Fig. S1a) and with tau burden in the global cortex, frontal, parietal, occipital, and posterior cingulate cortices, and amygdala in Aβ-positive individuals (see Supplementary Fig. S1b).

### Mediation of cognitive function between A/T/N neuroimaging biomarkers and odor identification ability

**Figure 4:**
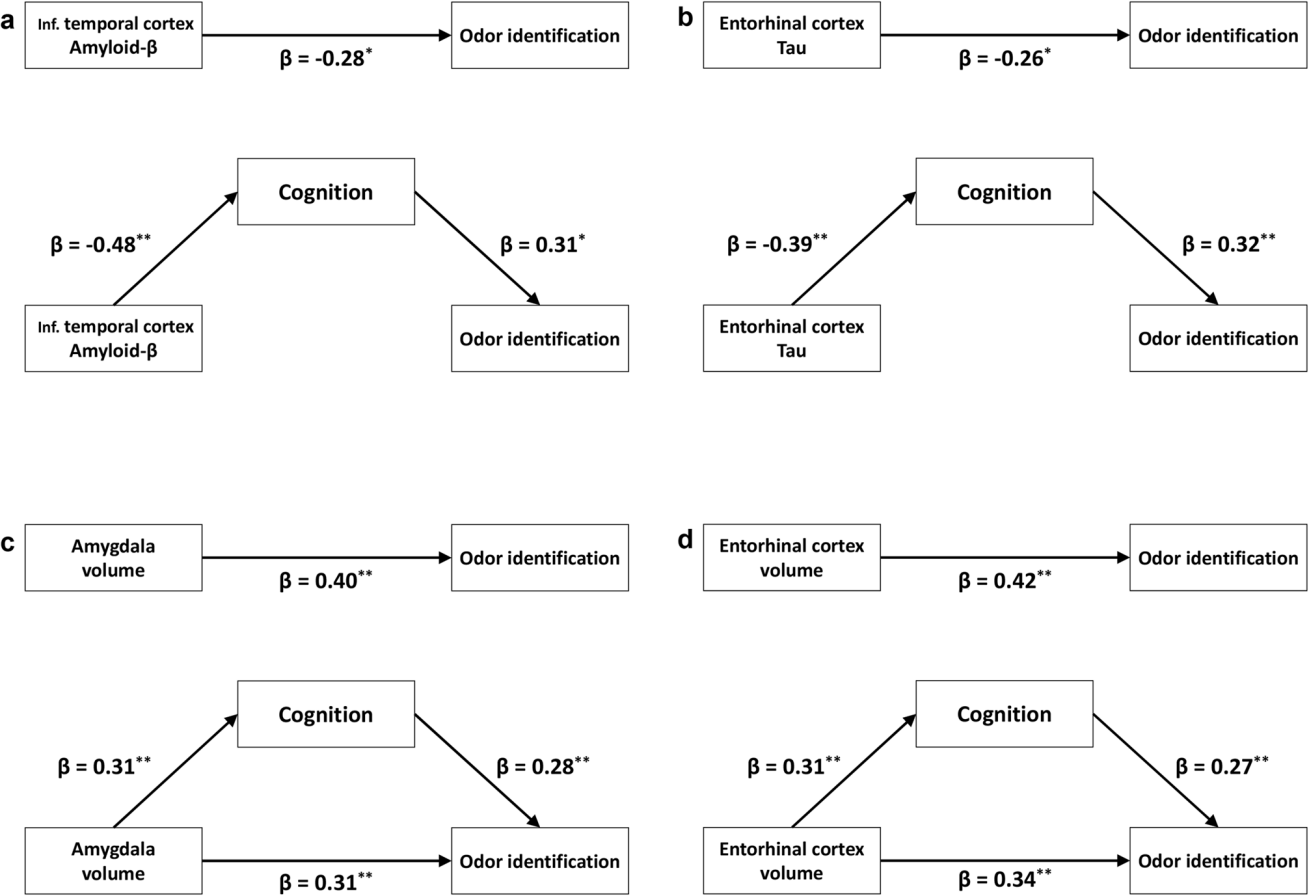
Schematic diagram of the path analyses for effect of A/T/N imaging biomarkers on odor identification ability. Path analysis for odor identification ability was performed using ^18^F-florbetaben, ^18^F-flortaucipir SUVR and cortical volume as predictors and the total cognition score as a mediator. In the upper part, total effects (direct effect + indirect effect) of the A/T/N imaging biomarkers on odor identification are shown. In (**c**, **d**), the lower side of the triangle represents the direct effects of the cortical volume on odor identification ability. Indirect effects of the A/T/N imaging biomarkers on odor identification ability are obtained by multiplying two β-coefficients between A/T/N imaging biomarkers and cognition, and between cognition and odor identification ability. Only significant β-coefficients are shown. **P < 0.001, *P < 0.05.

Path analysis was used to investigate whether the effect of A/T/N biomarkers on odor identification ability was directly or indirectly mediated by cognitive function. Volumes of the amygdala and entorhinal cortex showed direct effects on the CCSIT score, and an indirect effect mediated by total cognition score was also noted (Fig. 4c,d). In contrast to volume, regional Aβ and tau burden did not show direct effects on the CCSIT score in the regions that showed high significance in correlation analyses (Fig. 4a,b). The effect sizes of indirect effects mediated by total cognition score were relatively small for the regional cortical volumes, compared to the regional Aβ and tau burden.

## Discussion

In this study, we found that the hyposmia group exhibited smaller volume in the entorhinal cortex than the normosmia group. In Aβ-positive individuals, the hyposmia group exhibited more prominent volume atrophy in the medial and lateral temporal cortices than the normosmia group. In addition, deterioration of odor identification ability correlated with the volume atrophy in the medial and lateral temporal cortices. Meanwhile, there were no group differences in Aβ or tau burden, which did not correlate with the odor identification ability. Finally, of the three imaging biomarkers, only regional volume was directly related to the odor identification ability, while Aβ and tau burden were indirectly related to odor identification ability with cognitive dysfunction as a mediator.

Previous PET imaging studies have shown a higher negative predictive value of odor identification ability on Aβ burden in the elderly, suggesting that individuals with preserved odor identification ability are less likely to have Aβ deposit^9,10^. In contrast, another PET study showed a correlation between odor identification ability and regional Aβ burden in the posterior cingulate, temporoparietal, and lingual cortices in MCI and AD patients^22^. In a postmortem study, antemortem odor identification ability was negatively correlated with the pathological Aβ burden^7^. In contrast to these studies which did not control for the effect of cognitive function, our study demonstrated a lack of correlation between the burden and deterioration of odor identification ability, after controlling for total cognition score. Likewise, there was an indirect effect of Aβ burden on the odor identification ability mediated by cognition in the path analysis. These results indicate that Aβ burden has little direct effect on the odor identification ability.

Olfactory information is transmitted from the olfactory bulb to the primary olfactory cortex encompassing the anterior olfactory nucleus, piriform cortex, anterior cortical nucleus of the amygdala, and entorhinal cortices. The cortical regions such as insula, hippocampus, and hypothalamus have neuronal connections with the primary olfactory cortex for the processing of olfactory information^23^. Neurofibrillary tangle (NFT) pathology is found in the olfactory bulbs of AD patients^8,24^ and the spatial distribution of NFT pathology in the early stage of AD overlaps the key regions for olfactory information processing such as the entorhinal cortex, hippocampus, and amygdala^25^. We therefore may expect a contribution of tau pathology to impaired olfactory function in AD. However, a CSF tau biomarker study^26^, and postmortem studies, did not find a correlation between tau burden and deterioration of olfactory function^27,28^. One postmortem study including cognitively unimpaired elderly and AD patients showed an association between odor identification ability and NFT pathology in the entorhinal cortex and hippocampus^7^. However, this correlation might be driven by AD patients with high NFT burden and worse cognition, because the study did not for control cognitive function. In our study, tau burden in the widespread cerebral cortex correlated with the deterioration of odor identification score, without adjusting for cognitive function. However, this correlation disappeared after the inclusion of cognitive function in the list of covariates. Similar to the results for Aβ, we found an indirect effect of tau burden on odor identification ability mediated by cognition in the path analysis.

Atrophy in the primary olfactory cortex and hippocampus is consistently found in individuals with impaired olfactory function in cognitively unimpaired^9^, amnestic MCI^12^, and AD groups^29^. In accordance with previous studies, we found that impaired odor identification ability was associated with volume atrophy in the medial and lateral temporal cortices even after controlling for cognitive function. In contrast to our expectation that AD pathology, especially tau burden, might explain the volume atrophy and impaired odor identification ability, we found no direct effect of Aβ or tau burden on odor identification. Therefore, impaired olfactory function in AD may be attributable to neurodegeneration in the regions related to olfactory processing rather than the Aβ or tau burden. However, we must consider the contribution of other types of pathologies in AD. Coexisting Lewy body-related pathology in up to 50% of AD patients is a potential candidate^30^. Lewy body-related pathology in the olfactory bulb and amygdala, which receives the olfactory projection is reported as a potential cause of olfactory dysfunction in neurodegenerative diseases^27,28^. Another potential candidate is TDP-43 pathology found in 30–70% of AD^31^. TDP-43 preferentially accumulates in the medial temporal cortex and even in the olfactory bulb in AD, and may cause deterioration of odor identification function^32^.

Our study was limited first by a relatively small sample size. Additionally, we used a brief odor identification test, with only 12 items, instead of using the more complex University of Pennsylvania Smell Identification Test (UPSIT) with 40 items. Even though these two tests were cross-validated^33^, and the brief odor identification test is being widely used across studies, UPSIT may provide more precise cut-off for dichotomizing normosmia and hyposmia groups. In addition, we did not test for odor detection. Although this is highly correlated with odor identification test^1^, additional information about the olfactory function at receptor level may enable more accurate localization of the origin of olfactory dysfunction in AD. Lastly, with the cross-sectional study design, we could not examine changes in biomarkers throughout disease progression.

In conclusion, we identified the effects of A/T/N imaging biomarkers of AD on the odor identification ability. While Aβ and tau burden may have an indirect effect on odor identification ability mediated by cognition, neurodegeneration in the cortical regions related to olfactory processes may have a direct effect on odor identification in AD. Therefore, odor identification ability may be a useful biomarker for the neurodegeneration in AD.

## Methods

### Participants

For this study, we included 129 individuals who completed the Cross-Cultural Smell Identification Test (CCSIT), neuropsychological test battery, genotyping for apolipoprotein E (ApoE), brain magnetic resonance (MR) imaging, and two PET scans (^18^F-florbetaben for Aβ and ^18^F-flortaucipir for tau) at the Memory Disorder Clinic in Gangnam Severance Hospital during the period from January, 2015 to September, 2018. All patients with dementia (DEM) first presented with memory impairment and were clinically diagnosed with probable AD, as proposed by the National Institute of Neurological and Communicative Disorders and Stroke and the Alzheimer Disease and Related Disorders Association^34^. MCI patients were diagnosed according to the Petersen’s criteria based on their neuropsychological test performances^35^. No patient showed clinical features atypical of AD. Cognitively unimpaired individuals were healthy volunteers fulfilling Christensen’s diagnostic criteria^36^, and showing normal cognition in neuropsychological test. We excluded two participants who had a rhinological problem at the time of CCSIT or a history of rhinological surgery. Finally, 127 participants (40 cognitively unimpaired, 51 MCI, and 36 DEM) were included in this study. This study was approved by the institutional review board of Gangnam Severance Hospital (Ref# 3-2014-0286) and written informed consent was obtained from all participants and/or their legal guardians. All research was performed in accordance with the relevant guidelines and regulations.

### Olfactory assessment

Odor identification ability was assessed using the CCSIT^33^. The CCSIT includes 12 microencapsulated odorants attached to test cards. Participants were instructed to scratch, sniff, and select one of the 4 examples. The final score was calculated as the number of correct answers. Based on the resulting CCSIT scores, all participants were classified into either the normosmia (score ≥ 8) or hyposmia (score < 8) group, as previously reported^37^.

### Neuropsychological tests

All participants underwent the Seoul Neuropsychological Screening Battery^38,39^. We calculated subdomain scores for memory, language and related, visuospatial, frontal/executive, and attention functions with the items in the battery using the method described in a previous study^38^. Total cognition score was calculated as the sum of all subdomain scores. Mini-Mental State Examination (MMSE) and Clinical Dementia Rating sum-of-boxes (CDR-SB) scores were also assessed for global cognitive function.

### Acquisition of PET and MR images

Using a Biograph mCT PET/CT scanner (Siemens Medical Solutions, Malvern, PA, USA), we acquired PET images for 20 min at 80 min after the injection of ^18^F-flortaucipir, and at 90 min after the injection of ^18^F-florbetaben. The two PET scans were done in separate days. Computed tomography (CT) images were acquired for attenuation correction prior to the PET scan. Finally, 3D PET images were reconstructed in a 256 × 256 × 223 matrix with 1.591 × 1.591 × 1 mm voxel size using the ordered-subsets expectation maximization algorithm. Aβ-positivity was determined by the agreement of two nuclear medicine specialists using a validated visual assessment method^40,41^. Axial T1-weighted brain MR images were acquired by 3.0 T MR scanner (Discovery MR750; GE Medical Systems, Milwaukee, WI, USA) with 3D-spoiled gradient-recalled sequences (512 × 512 matrix with voxel spacing 0.43 × 0.43 × 1 mm voxel size).

### Image processing steps

T1-weighted MR images were processed with FreeSurfer 5.3 (Massachusetts General Hospital, Harvard Medical School; https://surfer.nmr.mgh.harvard.edu) software for creating participant-specific volume-of-interests (VOIs) mask images as described in our previous study^42^. MR images were first resliced to FreeSurfer space (256 × 256 × 256 matrix with 1 mm isovoxel) 25 segmented into gray and white matter, and then 3D-surfaces for gray and white matter were reconstructed. Cortical regions were parcellated using curvature information, and subcortical regions were segmented with the probabilistic registration method. By merging anatomically-related regions, participant-specific composite VOI images for 20 cortical and subcortical regions were created. Voxel counts for each region were considered to be regional volume.

Statistical parametric mapping 12 (Wellcome Trust Centre for Neuroimaging, London, UK) and in-house software implemented in MATLAB R2015b (MathWorks, Natick, MA, USA) were used to process the PET images and measure the regional uptake values. PET images were coregistered to individual MR images within FreeSurfer space, and then partial volume effect (PVE) was corrected with the region-based voxel-wise method by using the participant-specific VOI images^43^. PVE-corrected SUVR images were then created with the cerebellar crus median obtained by overlaying the template cerebellar crus VOI on the spatially normalized PET images. Finally, by overlaying the participant-specific composite VOI, we measured regional PVE-corrected SUVR values.

### Statistical analysis

SPSS 23 (IBM Corp., Armonk, NY, USA) was primarily used for the statistical analysis. For between-group comparisons, two-sample *t*-tests, and chi-square tests were used for continuous and categorical demographic data, respectively. Using the general linear model, regional SUVR values were compared between the normosmia and hyposmia groups after adjusting for age, sex, years of education, presence of ApoE ε4, and total cognition score. For the comparison of regional cortical volumes between the two groups, total intracranial volume was added to the list of covariates. For the correlation analysis between CCSIT scores and regional SUVR values, we first obtained standardized residuals of CCSIT scores and regional SUVR values after adjusting for age, sex, years of education, presence of ApoE ε4, and total cognition score by using the multiple linear regression model. For correlation analysis between CCSIT scores and regional cortical volumes, we used total intracranial volume as additional covariate for obtaining the standardized residuals. Pearson’s correlation was then used to test for correlation between the standardized residuals of CCSIT scores and those of regional SUVR and cortical volume. We repeated all tests without controlling for total cognition score. Only the regions that survived after correcting for multiple comparisons with the Benjamini-Hochberg’s false-discovery rate (FDR) method were considered to be significant^44^. To evaluate whether cognition mediates the effects of A/T/N imaging biomarkers on CCSIT scores, path analysis was performed using R Statistical Package (Version 3.6.1; Institute for Statistics and Mathematics, Vienna, Austria) using the regional SUVR and cortical volume as predictors and total cognition score as a mediator. Brain regions showing the highe significance for correlation were included in the analysis. Bootstrapping mediation analysis with 1,000 repetitions was used in pathway analyses^45^.

## Supplementary information

Supplementary figure 1.

## Data Availability

Data generated by this study are available from the corresponding author on reasonable request. The data are not publicly available due to privacy restriction.
