# Supplementary material for: Effect of A/T/N imaging biomarkers on impaired odor identification in Alzheimer's disease
Source: Sci Rep. 2020 Jul 14;10:11556. doi: 10.1038/s41598-020-68504-2 (PMC7360607; doi:10.1038/s41598-020-68504-2)
Supplement: Supplementary file 1 — Supplementary figure 1. [file 41598_2020_68504_MOESM1_ESM.doc]

**[Supplementary information]**

**Effect of A/T/N imaging biomarkers on impaired odor identification in Alzheimer's disease**

Min Seok Baek, MD,^1, a^ Hanna Cho, MD, PhD,^1, a^ Hye Sun Lee, PhD,^2^

Jae Hoon Lee, MD, PhD,^3^ Young Hoon Ryu, MD, PhD,^3^ Chul Hyoung Lyoo, MD, PhD^1*^

^1^Department of Neurology, Gangnam Severance Hospital, Yonsei University College of Medicine, Seoul, South Korea

^2^Biostatistics Collaboration Unit, Yonsei University College of Medicine, Seoul, South Korea

^3^Department of Nuclear Medicine, Gangnam Severance Hospital, Yonsei University College of Medicine, Seoul, South Korea

^a^Two authors equally contributed to this study.

**Correspondence:**

Chul Hyoung Lyoo, M.D., Ph.D.

Professor

Department of Neurology

Gangnam Severance Hospital

Yonsei University College of Medicine

20 Eonjuro 63-gil, Gangnam-gu,

Seoul, South Korea

Email: lyoochel@yuhs.ac

Tel: +82-2-2019-3326

Fax: +82-2-3462-5904


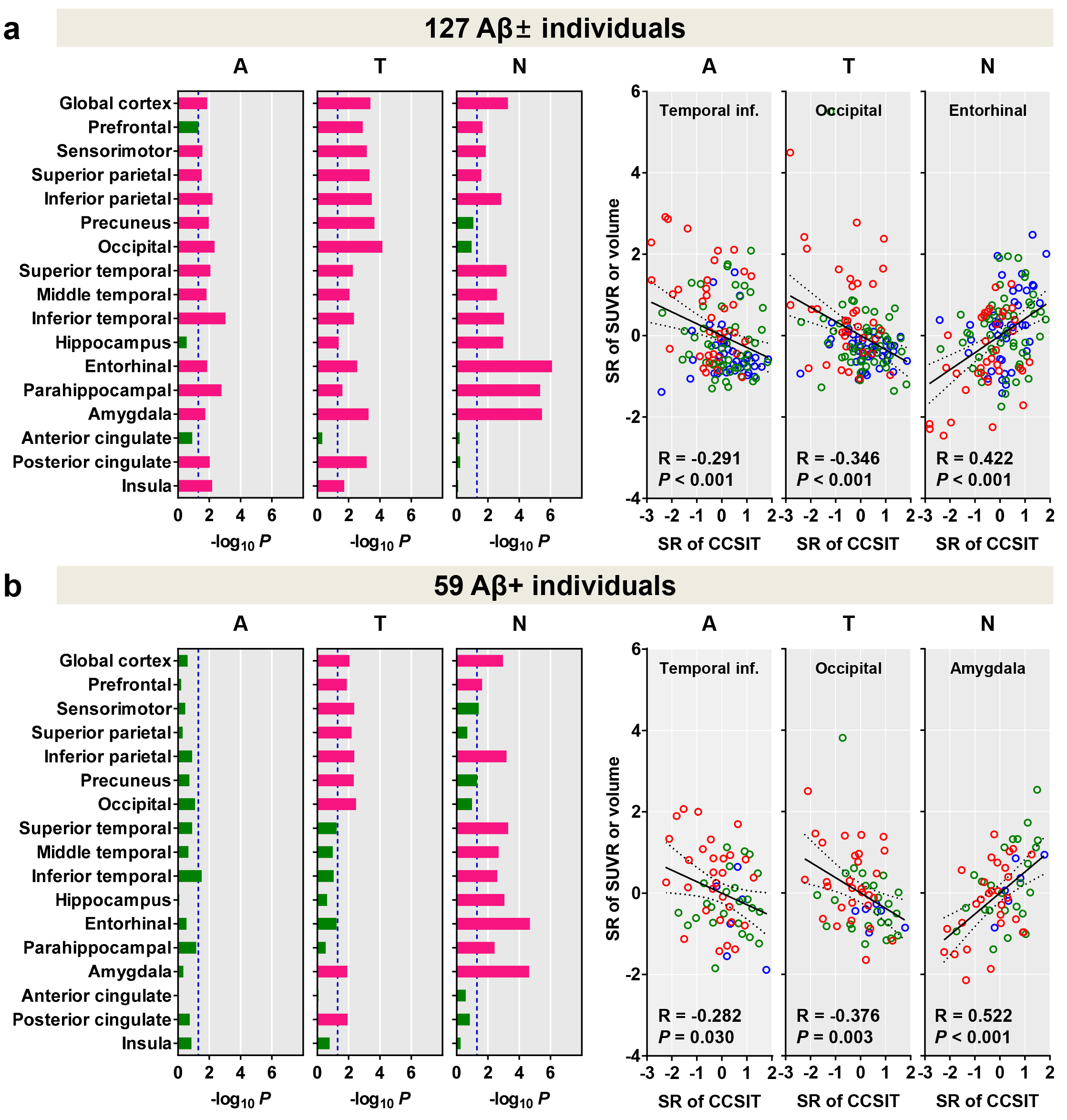


**Supplementary Fig. S1**. Correlation of CCSIT score with ^18^F-florbetaben, ^18^F-flortaucipir SUVR and cortical volume without correction for total cognition score.

We used Pearson’s correlation between the standardized residuals of CCSIT scores and A/T/N biomarkers obtained with multiple linear regression model after adjusting for age, sex, years of education, and presence of ApoE ε4. A covariate of intracranial volume was additionally adjusted for cortical volume. Horizontal bars represent *P*-values as -Log_10_*P.* Red bars represent the regions that survived correcting for region-wise multiple comparisons (false discovery rate-corrected *P* < 0.05), and blue dotted lines represent uncorrected *P* < 0.05.

In the right side panel, brain regions showing highest significance in correlation with ^18^F-florbetaben, ^18^F-flortaucipir and cortical volume were presented respectively. Blue dots represent CU participants, orange dots represent MCI patients, and red dots represent DEM. Abbreviations: Aβ+/- = Aβ-positivity, SUVR = standardized uptake value ratio, A = ^18^F-florbetaben SUVR, T = ^18^F-flortaucipir SUVR, N = cortical volume, SR = Standardized residual, CCSIT = Cross-Cultural Smell Identification Test, CU = cognitively unimpaired; MCI = mild cognitive impairment; DEM = dementia, ApoE = apolipoprotein E
